# Few-shot segmentation with duplex network and attention augmented module

**DOI:** 10.3389/fnbot.2023.1206189

**Published:** 2023-06-21

**Authors:** Sifu Zeng, Jie Yang, Wang Luo, Yudi Ruan

**Affiliations:** ^1^School of Economics and Management, Chongqing Jiaotong University, Chongqing, China; ^2^School of Information Science and Engineering, Chongqing Jiaotong University, Chongqing, China; ^3^College of River and Ocean Engineering, Chongqing Jiaotong University, Chongqing, China

**Keywords:** few-shot segmentation, semantic segmentation, mixture models, duplex mode, attention module

## Abstract

Establishing the relationship between a limited number of samples and segmented objects in diverse scenarios is the primary challenge in few-shot segmentation. However, many previous works overlooked the crucial support-query set interaction and the deeper information that needs to be explored. This oversight can lead to model failure when confronted with complex scenarios, such as ambiguous boundaries. To solve this problem, a duplex network that utilizes the suppression and focus concept is proposed to effectively suppress the background and focus on the foreground. Our network includes dynamic convolution to enhance the support-query interaction and a prototype match structure to fully extract information from support and query. The proposed model is called dynamic prototype mixture convolutional networks (DPMC). To minimize the impact of redundant information, we have incorporated a hybrid attentional module called double-layer attention augmented convolutional module (DAAConv) into DPMC. This module enables the network to concentrate more on foreground information. Our experiments on PASCAL-5i and COCO-20i datasets suggested that DPMC and DAAConv outperform traditional prototype-based methods by up to 5–8% on average.

## 1. Introduction

Deep convolutional neural networks have made significant strides in semantic segmentation. However, most high-performing models require a large number of pixel-level annotated training images. This annotation process is not only expensive but is also cumbersome, thereby posing challenges in obtaining enough samples in some scenarios. Consequently, achieving generalization across different scenarios becomes challenging. In light of this, few-shot learning, which aligns more closely with cognitive learning, is likely to become the primary focus of deep learning in the future. Few-shot segmentation involves the use of a learned feature representation from training images to segment a query image. However, this task remains a challenge when the object category falls outside the sample range and a significant variation in appearance and pose exists between the objects in the support and query images.

Shaban et al. (2017) contributed an initial approach to semantic segmentation with few samples and introduced the concept of “prototype.” Prototype-based methods are currently considered advanced in few-shot learning. This approach emphasizes the weight vector, which is computed through global average pooling guided by the ground truth mask in the embedded feature map. This vector effectively condenses discriminative information across feature channels, making it easier to compare features between support and query images for semantic segmentation.

However, many challenges are still encountered in the research of few-shot segmentation. The use of a single prototype for few-shot learning can result in semantic ambiguity and deteriorate feature distribution. Relying solely on a single prototype and simple operations for prediction can result in loss of inherent object details in the query image. Additionally, when large variation in appearance or scale of the object in few-shot learning is observed, making predictions based solely on support information can become difficult. Furthermore, the segmentation failure of ambiguous boundaries is also an existing problem in the few-shot segmentation task at this stage.

Recent advancements in techniques, such as feature boosting, prototype alignment, and iterative mask refinement, have addressed the aforementioned challenges effectively. CANet (Zhang et al., 2019) employs an iterative optimization module to merge query and support features in an optimized manner. Prototype mixture models (PMMs) (Yang et al., 2020) combine prototype mixture and duplex manner to fully exploit channel semantic and spatial semantic information. SCL (Zhang et al., 2021) utilizes a self-guided mechanism to generate an auxiliary feature prototype. ASGNet (Li et al., 2021) is designed to adaptively partition the support features into multiple feature prototypes and subsequently select the most relevant prototype for matching with the query image. CRCNet (Liu et al., 2022) presents a solution to address semantic ambiguity and feature distribution issues by introducing cross reference. This approach involves multiple interactions between support sets and query sets to improve their overall performance. However, these approaches become extremely fragile in terms of segmentation capability when facing more complex situations, such as ambiguous boundaries in few-shot segmentation tasks. When solving problems in ambiguous boundaries, starting with just the foreground can be challenging. The effective utilization of the duplex network and thorough mining of information allows the model to establish stronger relationships between the support and query sets with minimal samples, ultimately leading to improved segmentation accuracy.

**Figure 1:**
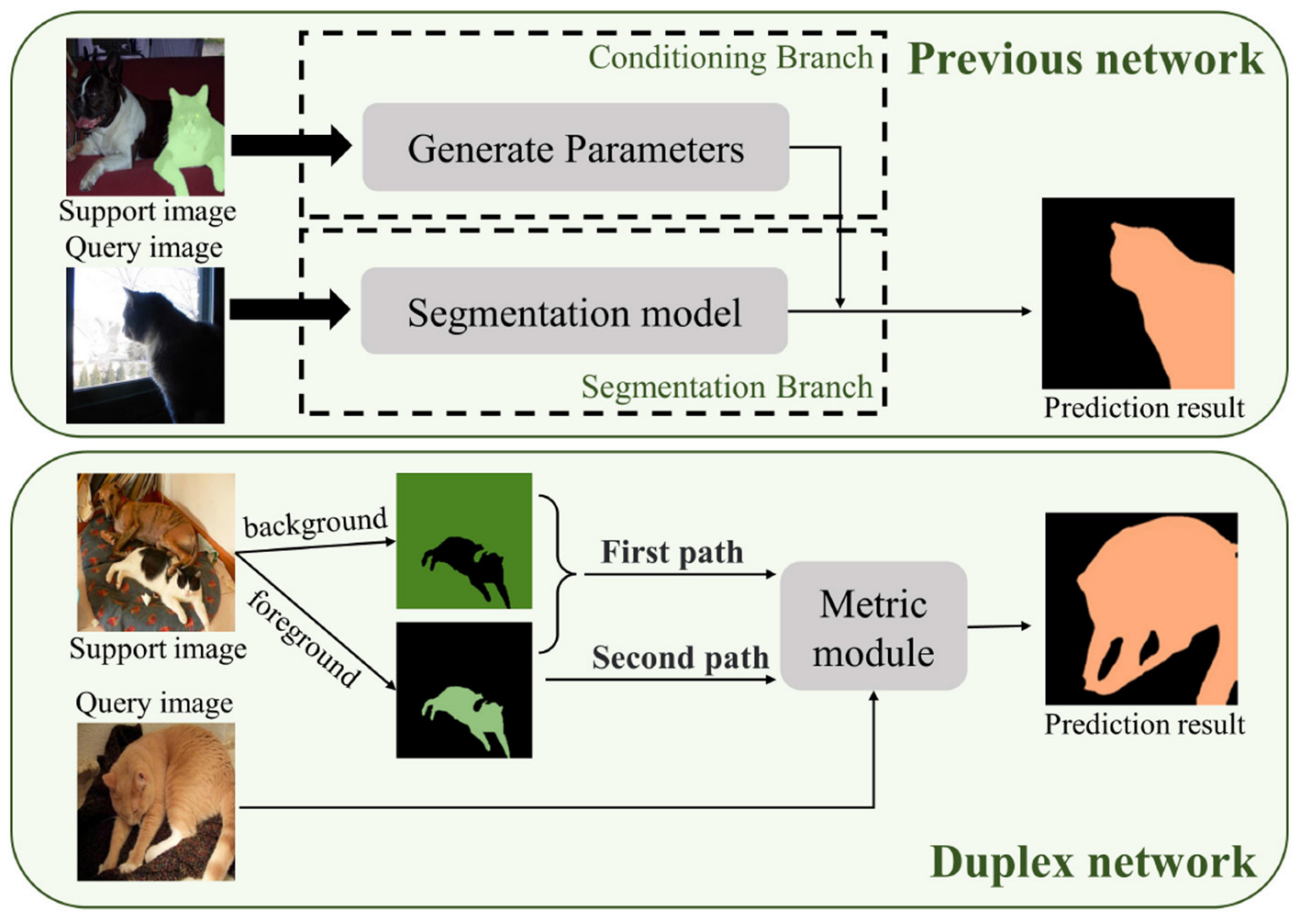
Visualization of duplex networks and previous networks.

Our research draws inspiration from the foreground–background and duplex modes utilized in PMMs. By utilizing the duplex mode, we can effectively utilize channel semantic and spatial semantic information to its fullest potential, as shown in Figure 1. This approach can enhance the accuracy of the image segmentation process in complex scenarios where the foreground and background have similar characteristics. However, we observed that the duplex mode in PMM only utilizes features that are extracted from the backbone network, indicating that the full potential of this mode remains untapped. Additionally, in-depth research on this mode is lacking in current studies. To gain a deeper understanding of the duplex manner, we plan to develop a new attention model and enhance the existing duplex mode through further investigation and exploration.

In this paper, we propose a novel approach called dynamic prototype mixture convolutional network (DPMC) inspired by the baseline method. Our method improves the duplex strategy used in the baseline by incorporating a prototype match structure to fully exploit the information in the support and query images. Additionally, we use channel information and spatial semantic information to segment the query image. To achieve sufficient support–query interaction, we introduce dynamic convolution in DPMC. Specifically, we apply kernel generation to produce different convolution kernels, which are applied with convolutions of different receptive fields to extract more image information. To enhance the segmentation performance of DPMC, we designed a double-layer attention augmented convolutional module (DAAConv). This module efficiently acquires contextual information, focuses on important regions, and removes redundant information. The attention module designed in this work effectively improves DPMC's ability to focus on the foreground, which results in enhanced segmentation performance. In conclusion, our experiments on the Pascal and COCO datasets have shown that the combination of DAAConv and DPMC significantly improves the baseline. Additionally, we conducted ablation experiments, which demonstrate that DAAConv enhances the duplex mode and DPMC outperforms the baseline.

The main contributions of our work are summarized as follows:

1. DPMC, which utilizes a duplex approach of suppressing the background and emphasizing the foreground, is presented in this study. Specifically, our proposed method is effective for addressing complex segmentation tasks with indistinct boundaries.
2. To improve the performance of duplex mode, DAAConv has been designed. This module can efficiently obtain contextual information and focus on important regions, ultimately enhancing the overall efficiency of the duplex mode.
3. The use of DAAConv and DPMC together fully maximizes the potential of the duplex concept. This approach achieves excellent performance in the classical dataset of few-shot learning, thereby significantly outperforming existing techniques.

The remainder of this paper is structured as follows: Section 2 reviews related works in semantic segmentation, attention and self-attention, and few-shot segmentation. Section 3 describes the DAAConv and DPMC models we constructed in detail. Section 4 demonstrates the superiority of our model through adequate experiments and proves the validity of our constructed model through multiple sets of ablation experiments. Section 5 summarizes our work and provides an outlook for the future.

## 2. Related work

In this section, we will discuss three aspects of work that are highly relevant to our work, including semantic segmentation, attention and self-attention mechanisms, and few-shot segmentation tasks.

### 2.1. Semantic segmentation

Semantic segmentation aims to divide an image into regions of different semantic categories. Classical methods, such as UNet (Ronneberger et al., 2015), correspond to fully convolutional networks with a U-shaped structure and symmetric encoding and decoding paths, as proposed by Ronneberger et al. It is not only known for its excellent segmentation accuracy but also for its decent speed. Other methods, such as PSPNet (Zhao et al., 2017) and DeepLab (Chen et al., 2017a,b), are also based on fully convolutional networks (FCN; Long et al., 2015). However, Their common shortcoming is limited ability to gain long-range context information, missing the global information. Recent research has focused on how to widen the visual field to simulate the remote context of an image. Inspired by non-local (Wang et al., 2018) approaches, some methods (Chen et al., 2016; Liu et al., 2017; Ding et al., 2018; Li et al., 2019; Hou et al., 2020; Pal et al., 2022) use attentional mechanisms to establish connections between image contexts. Transformer architectures also achieve good results in semantic segmentation, focusing on multi-scale feature fusion (Zhang et al., 2020; Chen et al., 2021; Wang et al., 2021; Xie et al., 2021; Jin et al., 2022a,b,c, 2023), and contextual feature aggregation (Liu et al., 2021; Strudel et al., 2021; Yan et al., 2022). For example, SETR (Zheng et al., 2021) uses the transformer framework to serialize images to achieve a fully attention-based feature representation encoder. In Cross ViT (Chen et al., 2021), a dual-branch transformer is used to group patches of different sizes in images, and multiple interactions with the attention mechanism are performed to integrate information better. FPANet (Wu et al., 2022) utilized a lightweight feature pyramid fusion module FPFM to reduce the number of feature channels. Additionally, SeBiFPN was employed to acquire semantic and spatial information from images and to merge features from various levels.

### 2.2. Attention and self-attention mechanisms

The introduction of the attention mechanism has shifted the attention to important areas and ignored irrelevant parts. The application of attention mechanism can be regarded as a dynamic selection process that adaptively achieves feature weighting processing based on the importance of the input. The superiority of the attention mechanism has been demonstrated in multiple visual tasks. For example, in semantic segmentation tasks, the classic channel attention module called SENet (Hu et al., 2018) improves the representation ability of the network by modeling the interdependence among convolutional feature channels. Classic spatial attention module (SAM) can also be utilized (Zhu et al., 2019). In recent years, many hybrid attention modules, such as the convolutional block attention module (CBAM; Woo et al., 2018), which contains the channel attention module (CAM) and the spatial attention module (SAM). For instance, DANET (Fu et al., 2019) employs two distinct attention modules in the spatial and channel dimensions and combines the outputs of these modules to enhance feature representation, thereby effectively improving segmentation accuracy. MANet (Wang et al., 2022) is used to alleviate the problem of excessive complexity of non-local networks by replacing the traditional single densely connected graph with two sparsely connected graphs. Attention mechanisms have many types, and excellent hybrid attention mechanisms similar to CBAM and DANET have not yet been fully developed.

Self-attention mechanisms and non-local neural networks have been proven to be highly successful in various tasks because of their effectiveness in modeling long-range contextual information. Particularly, within the realm of natural language processing tasks, self-attention mechanisms can automatically calculate and explore the relationships between the sentences themselves and finally obtain the connections among each variable in the sentence and all variables. For example, in transformer, self-attention helps to encode specific words while still obtaining information from other words in the sentence. However, in the field of imaging, the mechanisms for paying attention have not been sufficiently developed. In image classification tasks, Bello et al. (2019) developed a novel two-dimensional relative self-attention mechanism, which infuses relative positional information while maintaining translational equivariance, thereby making it very suitable for images. This attention mechanism is used to improve the convolutional operator to replace convolution by concatenating convolutional feature maps with a set of feature maps generated by the self-attention mechanism. The construction of the attention mechanism in this paper is also inspired by this.

### 2.3. Few-shot segmentation

Manual annotation is time consuming, laborious, expensive, and does not fit the learning style of humans. Therefore, been studied extensively in recent years. Existing few-shot learning updates these three components by incorporating two steps: first, associating the encoder's support set and query set image features, and second, minimizing the loss of the difference between the measurement prediction and the underlying facts of the query sample. A prototype learning or feature stitching approach is adopted when we need to solve the issue about how to associate support and query images. In OSLSM (Shaban et al., 2017), a two-branch one-time semantic image segmentation method is introduced to achieve few-shot segmentation. In this method, the first branch takes the labeled image as an input and produces a vector of parameters as an output. The second branch takes these parameters and a new image as input and produces a new class of image segmentation masks as output. PL (Dong and Xing, 2018) uses a prototype network to learn a prototype for each class. Then, it computes the cosine similarity between the test sample and each prototype to predict the class label. In CANet (Zhang et al., 2019), an iterative optimization module is used to iteratively optimize the results for merged queries and supporting features. In PMMs, the proposed model enhances the representations of semantic information in images through the association of image regions with multiple prototypes. Using an expectation-maximization (EM) algorithm, the model estimates the prototype-based semantic representation. Interestingly, PMMs use duplex mode to suppress the background region. Although the simple duplex network can partially address the problem in ambiguous boundaries, the limited interaction between the support set and query set, as well as the lower exploitation of the duplex network, can negatively influence the performance of PMM. In SSA-Net (wang et al., 2022), a spatial self attention network is introduced to broaden the sensory domain and enhance representation learning by extracting valuable contextual information from deeper layers through the use of a self-attention mechanism. CRCNet (Liu et al., 2022) explains the concept of cross reference, which involves predicting and cross-referencing query images and support images simultaneously. This approach helps mitigate issues related to semantic ambiguity and feature distribution that arise during few-shot learning. However, CRCNet ignores the deeper mining of both when pursuing a large number of interactions between support and query sets.

Our study is inspired by the duplex manner in PMMs, which can effectively depress background regions in few-shot segmentation tasks and improve the accuracy of segmentation. Features extracted through the backbone network, such as Resnet, contain a significant amount of redundant information. Despite their effectiveness in capturing local details, these features often fail to provide a global information of the input data. This limitation arises from the relatively narrow perceptual field of the network, which hinders the extraction of more comprehensive and meaningful information. In light of these observations, we believe that further exploration of feature selection and representation techniques is necessary to improve the performance of deep learning models in complex tasks. Therefore, the information extracted from the backbone network should be further processed before using the duplex method to maximize the effectiveness of the method. We have also made appropriate improvements to the duplex manner in PMMs to make the support–query interaction more adequate.

## 3. Method

### 3.1. Overview

**Figure 2:**
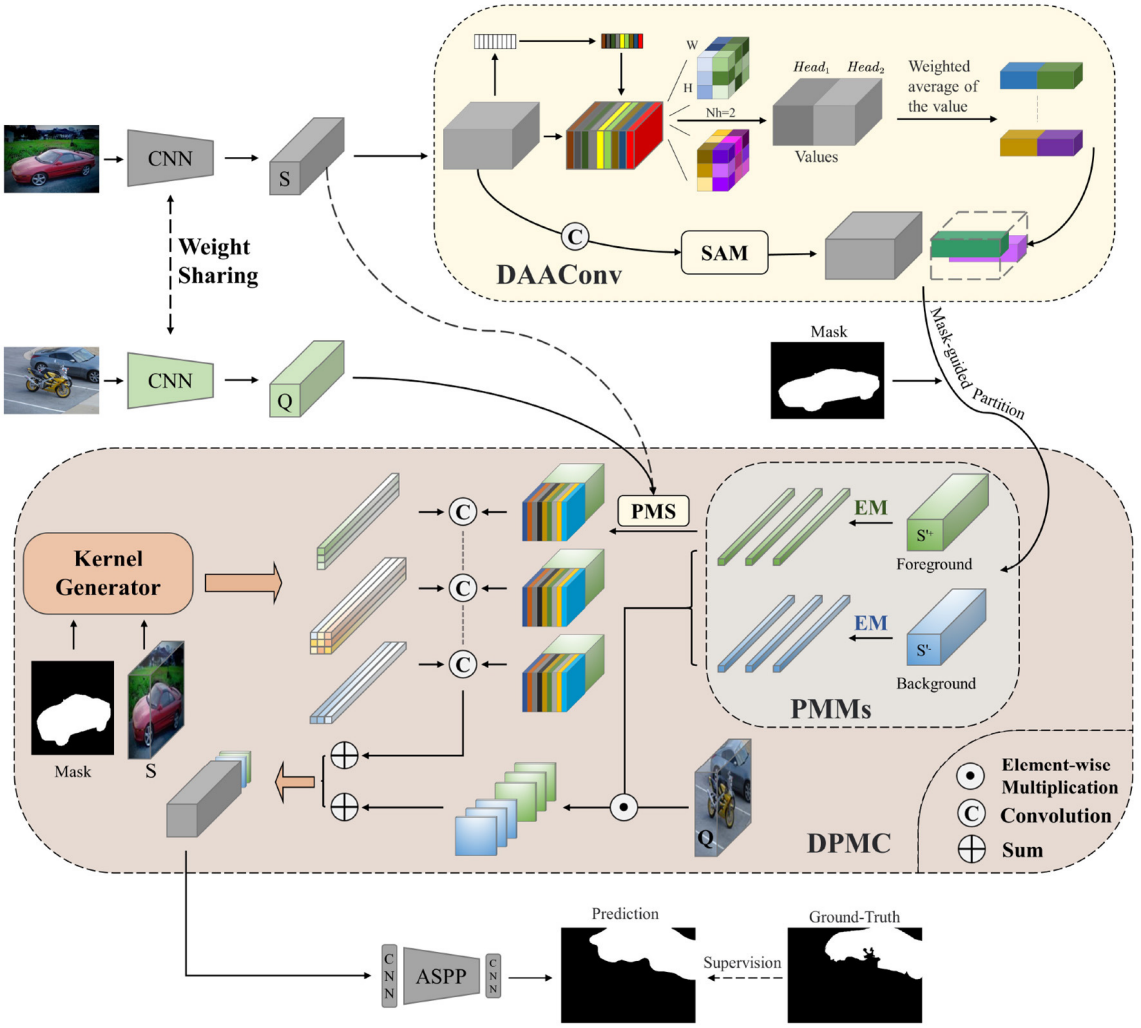
Overall structure of our method with the double-layer attention augmented convolutional module (DAAConv) and the dynamic prototype mixture convolutional network (DPMC).

To acquire more contextual information within the learning network, extract the target regions efficiently, and play the role of duplex mode efficiently, we design DAAConv, as shown in Figure 2.

Our model also includes two network branches: the support branch and the query branch. Two weight-sharing CNNs are used as the backbone network for feature extraction in the support and query branches. The support image's feature set S is then fed into DAAConv. After being processed by the attention module, the feature set continues to be fed into the DPMC. In DPMC, the feature set is first divided into a positive (foreground) sample set, S+, and a negative (background) sample set, S-. Subsequently, a Prototype vector is generated using the EM algorithm before proceeding to the next step with duplexing. One side of the duplex mode uses PMS to activate query features, and dynamic convolution using custom convolution kernels learned from the support set by the kernel generator, which will effectively connect the support and query sets, while the other side generates probability maps by element-wise multiplication. Finally, the two sides are combined for semantic segmentation.

In summary, we construct a new hybrid attention module called DAAConv and a new duplex network called DPMC. The two modules combined in the network can effectively obtain contextual information, focus on important regions, improve the duplex model performance, fully mine the information in support and query, and increase the support–query interaction. The complementarity of the two modules effectively addresses the lack of support–query set interaction and deeper information mining in traditional few-shot segmentation. Next, we will explain each part mentioned above in detail.

### 3.2. DAAConv module

Next, we will formally introduce our DAAConv module. First, to obtain the channel information of the support set, we utilize the SE (Hu et al., 2018) attention module in the first layer of the attention mechanism, which mainly consists of squeeze and excitation, to effectively determine the meaning of each channel and weight the features according to the meaning, so as to highlight the important features and repress the non-important ones. The use of this module has successfully enabled the information to be used in various ways. The use of this module successfully focuses the information on the foreground part and weakens the background part.

Specifically, we refer to the height, width, and number of input filters of an activation map, given an input tensor S of shape (*H, W, C*). First, we pass X through the squeeze and excitation channel attention network. Then we obtain the output:


(1) $$\begin{array}{l} DAA_{1}\left(S\right)=U=SE\left(S\right). \end{array}$$

Next, we feed the output *U* ∈ ℝ^*H*^′ × *W*′ × *C*′ into our second layer of attention, the self-attention mechanism. For the choice of the second layer of the attention mechanism, we draw on the multi-head-attention (MHA) part of a novel attention mechanism (AAConv; Bello et al., 2019). Self-attention is a recent advancement in capturing long-range interactions, but is mainly used in sequence modeling and generative modeling tasks. In contrast, AAConv preserves translational isomorphism while injecting relative position information, hence making it well suitable for images. We only selected the multihead-attention part as our second stage of the attention mechanism:


(2) $$\begin{array}{l} DAA_{2}\left(U\right)=MHA\left(U\right). \end{array}$$

The composition of DAA (Double-layer Attention Augmented networks) can effectively enable features to obtain contextual information and focus attention where we need it. DAA is only a part of our double-layer attention augmented convolutional module.


(3) $$\begin{array}{l} DAA\left(S\right)=DAA_{2}\left(DAA_{1}\left(S\right)\right). \end{array}$$

In our experiments, we found that the improvement of segmentation accuracy is more limited if we only use DAA. DAA can effectively capture the long-distance information of an image but ignores the local information. So, we introduce an additional feature mapping in the network or the second layer of our two-layer attention module. We achieve a balance between long-range and close-range information by concatenating the convolution module, which enhances localization, with the self-attention module, which captures long-range information.

We pass the support sets extracted through the backbone network sequentially through the ordinary convolution and SAM (Zhu et al., 2019).


(4) $$\begin{array}{l} X^{\prime }=SAM\left(Conv\left(S\right)\right). \end{array}$$

Finally, we concatenate the additional feature map obtained and the attentional feature maps generated by DAA through the concatenating operation.


(5) $$\begin{array}{l} DAAConv\left(S\right)=S^{\prime }=Concat\left[SAM\left(Conv\left(S\right)\right),DAA\left(S\right)\right]. \end{array}$$

We solve the high memory footprint of the self-attentive mechanism by using smaller batch sizes.

### 3.3. DPMC networks

#### 3.3.1. Prototype generation

After the image features have passed through the DAAConv we have designed, more contextual information is effectively extracted, and important region features are automatically captured, which will be of good help for our next processing. We will then describe in detail the DPMC that we have designed.

We name the DAAConv(X) obtained above as *S*′ ∈ ℝ^*H*^″ × *W*″ × *C*″. S is spatially divided into foreground samples *S*^′+^ for object part learning and background samples *S*^′−^ for background part learning. In the prototyping section, DPMC relies on the idea of the probability mixture model (Yang et al., 2020), as


(6) $$\begin{array}{l} p\left(s_{i}^{\prime }|\theta \right)=\overset{K}{\underset{k=1}{\sum }}w_{k}p_{k}\left(s_{i}^{\prime }|\theta \right). \end{array}$$

where *w*_*k*_ represents weight, and $p_{k}\left(s_{i}^{\prime }|\theta \right)$ denotes the *k*^*th*^ base model.

Next, we obtain the prototype using the EM algorithm, which consists of iterative E-steps and M-steps. The expected value of the sample *s*_*i*_ is calculated in each E-step.


(7) $$E_{ik}=\frac{p_{k}\left(s_{i^{\prime }}|\theta \right)}{\sum_{k=1}^{k}p_{k}\left(s_{i^{\prime }}|\theta \right)}.$$

In each M-step, the mean vectors are updated using the expectation, as


(8) $$\begin{array}{l} \mu_{k}=\frac{\sum_{i=1}^{N}E_{ikS_{i}^{\prime }}}{\sum_{i=1}^{N}E_{ik}} \end{array}$$

We have successfully obtained the prototype using by EM algorithm. Then, we will use our duplex mode to process the prototype we obtained.

#### 3.3.2. Job in duplex mode

The prototype vector that corresponds to *S*^′+^ is $\mu^{+}=\left\{\mu_{k}^{+},k=1,\ldots K\right\}$, and the prototype vector corresponding to *S*^′−^ is $\mu^{-}=\left\{\mu_{k}^{-},k=1,\ldots ,K\right\}$. In the baseline, the authors have conducted ablation experiments, which demonstrate that the effect is optimal when “K = 3.” Therefore, we will not perform additional experiments and will use “K = 3” as the default value.

##### 3.3.2.1. PMS

**Figure 3:**
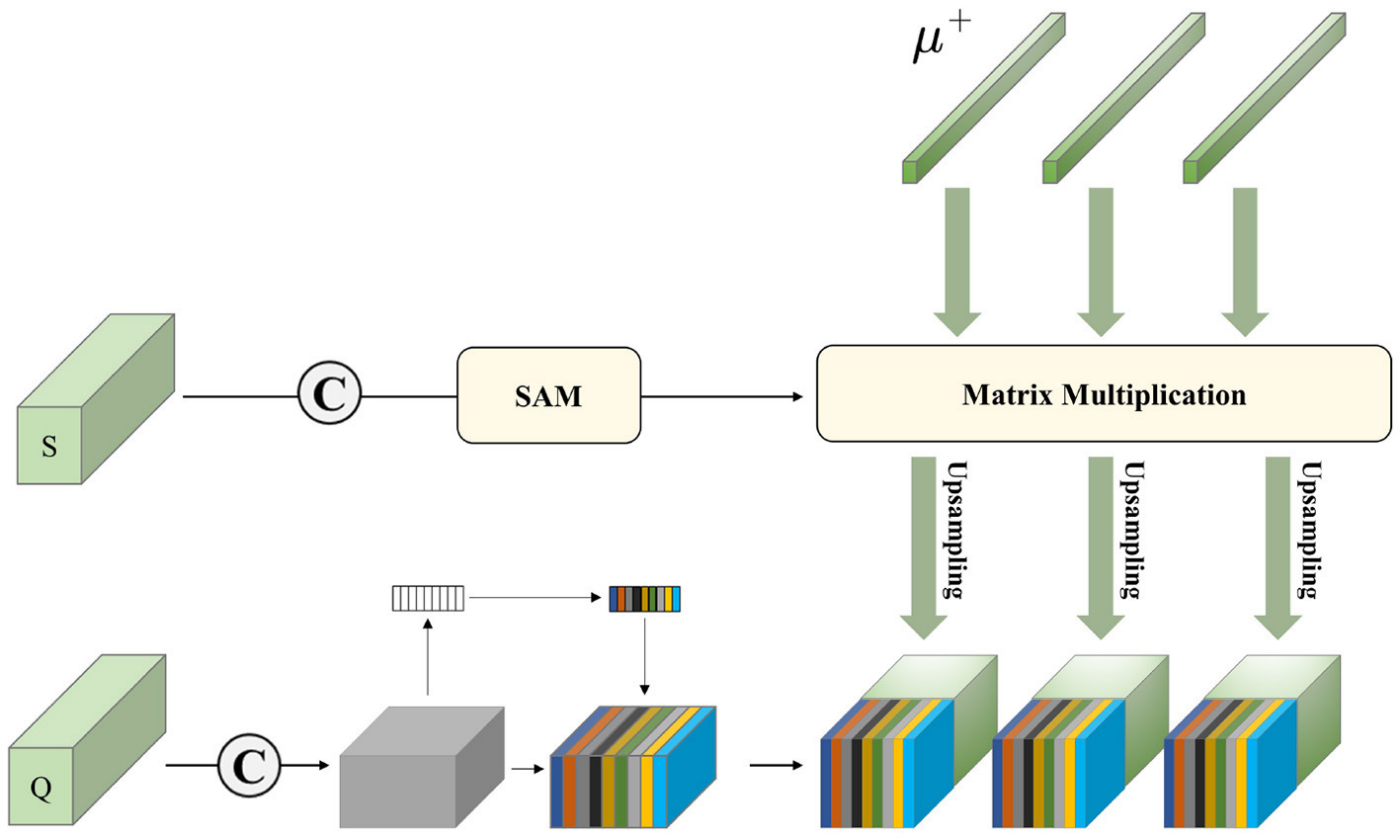
Visual illustration of our proposed PMS.

Distinguishing from the P-Match in baseline, we redesigned a PMS, as shown in Figure 3. We perform the Matrix Multiplication of the processed support set with the foreground prototype. The feature fusion of support sets at different scales can mine more information in the support set. We then upsample the obtained results into the query set processed by the SE module.


(9) $$\begin{array}{l} Q^{\prime }=PMS\left(\mu_{k}^{+},Q,S\right),k=1,\ldots ,K. \end{array}$$

Compared with baseline, the PMS we designed accomplishes a deeper mining of support set information by fusing features from different scales of support sets.

##### 3.3.2.2. Dynamic convolution

For more accurate segmentation, we innovatively introduce the dynamic convolution of the feature sets obtained from the EM algorithm and the PMS. The dynamic convolution generator based on the support set can enable more sufficient interaction between the support and query sets. Specifically, the support feature set S and its corresponding masks are inputted into a kernel generator that produces the dynamic convolution *ker*_1_, *ker*_2_ and *ker*_3_ (i.e., one set of quadratic kernels and two sets of asymmetric kernels). Then, for each of the three prototypes, we perform convolution operations and summation using each of these three convolution kernels.


(10) $$\begin{array}{l} Q_{k}^{\prime \prime }=Conv\left(ker_{k},PMS\left(\mu_{k}^{+},Q,S\right)\right)\text{,}k=1,\ldots ,K. \end{array}$$

More details about the kernel generator can be found in Liu et al. (2022).

#### 3.3.3. Another job in duplex mode

In this section, we first multiply each prototype vector by the query feature Q using Element-wise Multiplication. Consequently, the resulting graph is converted into a probability map by using the softmax function on the channels and summing them to produce two probability maps, foreground, and background, $M_{p}^{+}$, and $M_{p}^{-}$.

To activate the object of interest, this is then further concatenated with the query function:


(11) $$\begin{array}{l} Q^{\prime \prime \prime }=Concat\left(M_{p}^{+},M_{P}^{-},Q^{\prime \prime }\right). \end{array}$$

Finally, *Q*^‴^ is passed to a decoder to generate a segmentation mask *M*_*Q*_ for the query image:


(12) $$\begin{array}{l} M_{Q}=Conv\left(ASPP\left(Conv\left(Q^{\prime \prime \prime }\right)\right)\right). \end{array}$$

## 4. Experiments

### 4.1. Experimental setting

#### 4.1.1. Datasets

In our experiment, we validated the model on two classic few-shot segmentation datasets, namely, PASCAL-5^*i*^ and COCO-20^*i*^. The first dataset is generated from PASCAL VOC 2012 (Everingham et al., 2009) with additional mask annotations from SDS (Hariharan et al., 2014) and consists of 20 semantic categories evenly divided into four-folds. The second dataset is built from MS COCO (Lin et al., 2014) and is composed of 80 semantic categories divided into four folds. Notably, COCO-20^*i*^ includes 40,137 images (80 categories), which is higher than PASCAL-5^*i*^. Therefore, COCO-20^*i*^ is a more challenging benchmark.

#### 4.1.2. Evaluation indicators

In our experiments, we use mIoU as our evaluation metric. mIoU is a standard metric for semantic segmentation that measures the overlap ratio between the generated and original regions (i.e., the ratio of intersection to union). A higher mIoU indicates better segmentation results. mIoU can be calculated as follows


(13) $$\begin{array}{l} mIoU=\frac{1}{C}\overset{C}{\underset{i=1}{\sum }}IoU_{i}, \end{array}$$

(14) $$\begin{array}{l} IoU=\frac{TP}{TP+FP+FN}. \end{array}$$

In predicted masks, TP (true-positives) are pixels that are truly predicted to be a part of the class, FP (false-positives) are pixels that are falsely predicted to be a part of the class, and FN (false-negatives) are pixels that are falsely predicted not to be a part of the class.

#### 4.1.3. Implementation details

Our approach takes PMMs (Yang et al., 2020) as the baseline and employs VGG-16 and ResNet50 as the backbone. To obtain the prototype, we iterated the EM algorithm for 10 rounds. We use four data enhancement strategies (Zhang et al., 2019): normalization, horizontal flipping, random cropping, and random resizing. Although limited by computational resources, we used a learning rate of 0.0035 and a batch size of four to train both datasets, which did not affect our ability to demonstrate the effectiveness of our method. We ran a total of 200,000 steps. Our experiments were implemented using PyTorch 1.7 and ran on an NVIDIA RTX 3060 12g GPU.

### 4.2. Duplex mode analysis

**Figure 4:**
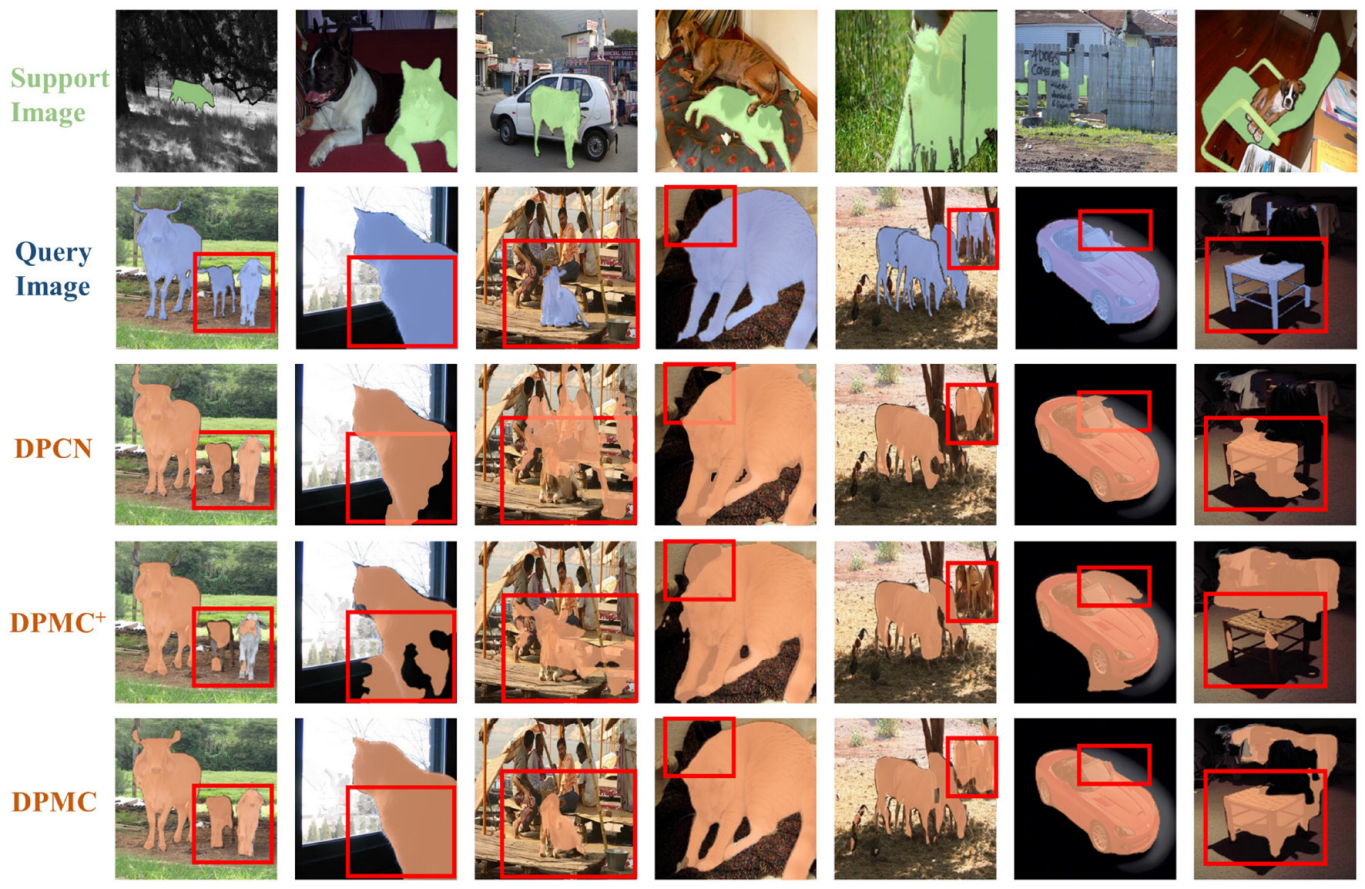
Segmentation results of DPCN, DPMC^+^, and DPMC. DPCN represents the method used in Liu et al. (2022). The method does not use duplex networks. DPMC^+^ represents the working path that only uses the foreground (i.e., the working path where the PMS is located). DPMC represents our complete duplex network.

Several existing studies have proposed models for solving the few-shot segmentation task using duplex networks. However, these models have only utilized duplex networks as a tool and have not delved into further exploration of their potential. This instance makes the performance of the duplex mode not fully developed. To demonstrate that duplex mode is a good solution for few-shot segmentation tasks, we visualize the segmentation results of DPMC with duplex mode, DPMC with only a single chain in the foreground, and DPCN with excellent performance without duplex mode, as shown in Figure 4. The single chain and DPCN can also perform the segmentation task well when segmenting images with a strong difference between the object and the background. However, when the background is more similar to the segmented objects, the duplex mode shows its superiority well, such as the chair and the cow. The much better-performing DPCN does not perform well with this tricky problem and show larger errors in two tasks, cow and chair, where the background is extremely similar to the segmentation target.

The experimental results in Table 1 show that the use of duplex mode effectively improves the segmentation accuracy by 3.5%.

**Table 1:** Duplex mode analysis of our DPMC on PASCAL-5^*i*^.

**Model**	**Mean**
DPMC^+^+DAAConv	58.4
DPMC+DAAConv	**61.9**

DPMC^+^ represents the working path that uses only the foreground (i.e., the working path where the PMS is located). The bold values represent the best performance.

### 4.3. Performance

PASCAL-5^*i*^: We report the mIoU in the 1-shot and 5-shot settings in Table 2. In 1-shot and 5-shot settings, they outperform state-of-the-art methods, especially for the 5-shot setting, with a backbone of ResNet50, exceeds the baseline by 7% and exceeds the previous best model HSNet by 0.2%. Our model also performs well in the 1-shot setting, thereby outperforming the baseline by 5.5%, HSNet by 2.2%, and MMNet by 0.1%. Our experimental results show that our model effectively improves the baseline and enhances the performance of the duplex mode.

**Table 2:** Comparison with state-of-the-arts on PASCAL-5^*i*^ dataset under **1-shot** and **5-shot** settings.

**Method**	**Backbone**	**1-shot**					**5-shot**				
		**Fold-0**	**Fold-1**	**Fold-2**	**Fold-3**	**Mean**	**Fold-0**	**Fold-1**	**Fold-2**	**Fold-3**	**Mean**
OSLSM (Shaban et al., 2017)	VGG16	33.6	55.3	40.9	33.5	40.8	35.9	58.1	42.7	39.1	43.9
co-FCN (Rakelly et al., 2018)	VGG16	36.7	50.6	44.9	32.4	41.1	37.5	50.0	44.1	33.9	41.4
HSNet (Min et al., 2021)	VGG16	59.6	65.7	59.6	54.0	59.7	64.9	69.0	64.1	58.6	64.1
PFENet (Tian et al., 2020)	ResNet50	61.7	69.5	55.4	56.3	60.8	63.1	70.7	55.8	57.9	61.9
SCL (Zhang et al., 2021)	ResNet50	63.0	70.0	56.5	57.7	61.8	64.5	70.9	57.3	58.7	62.9
MMNet (Wu et al., 2021)	ResNet50	62.7	70.2	57.3	57.0	61.8	62.2	71.5	57.5	62.4	63.4
CWT (Lu et al., 2021)	ResNet50	56.3	62.0	**59.9**	47.2	56.4	61.3	68.5	**68.5**	56.6	63.7
CRCNet (Liu et al., 2022)	ResNet50	63.4	69.7	55.8	56.9	61.5	65.2	70.9	55.9	61.8	63.5
MANet (Ao et al., 2022)	ResNet101	**63.9**	69.2	52.5	**59.1**	61.2	**66.7**	70.3	54.2	**64.5**	63.9
**RPMMs (Baseline) (Yang et al.**, **2020****)**	VGG16	47.1	65.8	50.6	48.5	53.0	50.0	66.5	51.9	47.6	54.0
**DPMC+DAAConv (Ours)**	VGG16	55.8	69.5	55.4	52.9	58.4	62.2	69.8	58.3	54.5	61.2
**RPMMs (Baseline) (Yang et al.**, **2020****)**	ResNet50	55.2	66.9	52.6	50.7	56.4	56.3	67.3	54.5	51.0	57.3
**DPMC+DAAConv (Ours)**	ResNet50	62.9	**70.7**	56.8	57.2	**61.9**	65.7	**71.9**	62.1	57.5	**64.3**

mIoU of each fold and averaged mIoU of all folds are reported. The baseline is RPMMs. The bold values represent the best performance.

**Figure 5:**
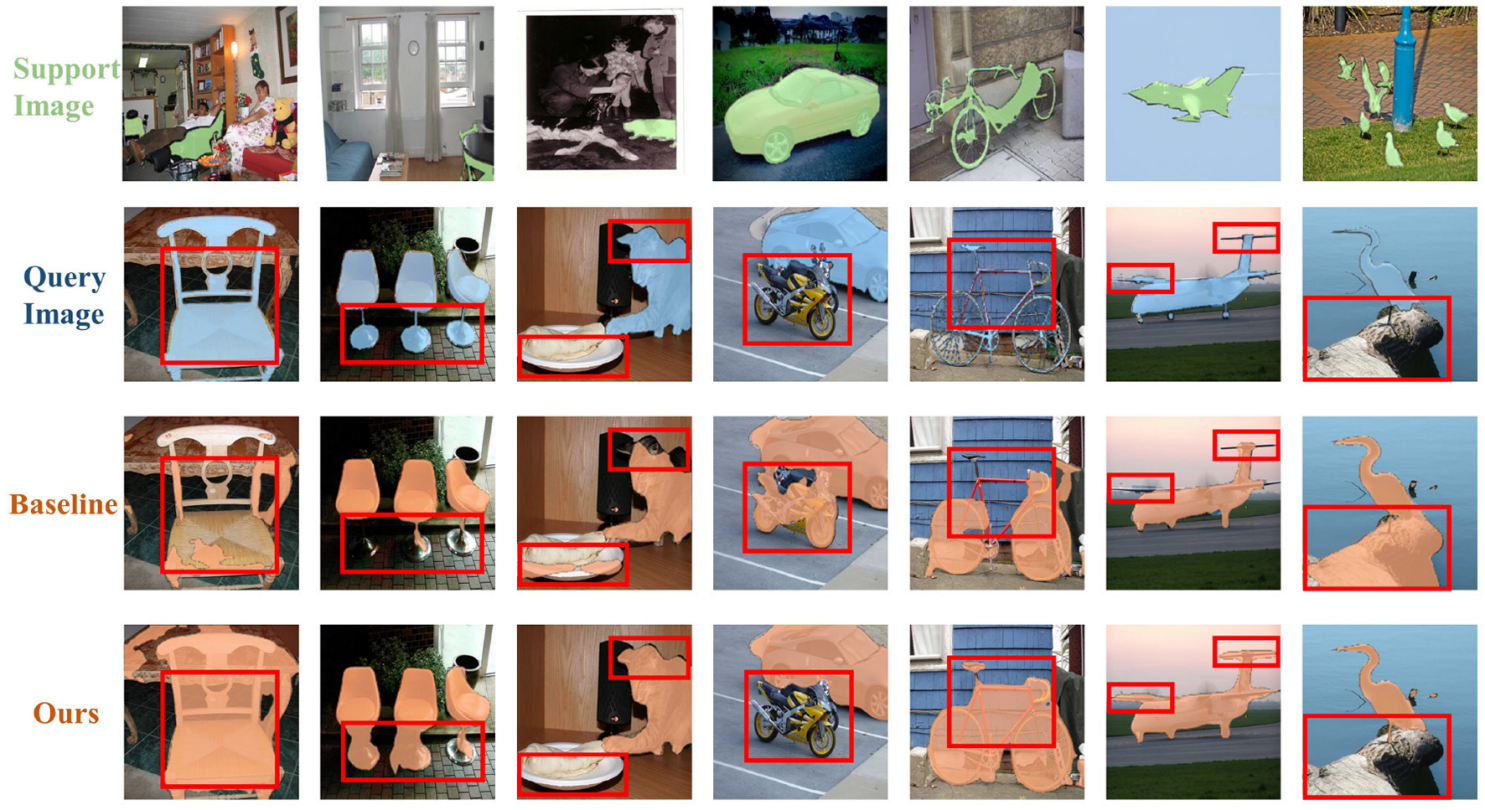
Segmentation results of our model and baseline.

We visualized several random segmentation results in the PASCAL-5^*i*^ dataset, as shown in Figure 5. Our network shows a significant improvement in segmentation compared with the baseline. We can also observed from the figure that our network can dig into finer details compared with baseline, as seen in places, such as stool legs and airplane wings. Our network can effectively distinguish and segment similar objects, such as motorbikes and cars, when they appear together, thus outperforming the baseline.

COCO-20^*i*^: COCO-20^*i*^ is more challenging as it has a larger variety of objects and greater variation than PASCAL-5^*i*^. Our model performs well in 1-shot and 5-shot settings. Table 3 reports the mIoU of our model in these settings, showing that our model significantly outperforms the baseline. Our model outperforms the baseline by 8.1% in the 1-shot setting and by 7.2% in the 5-shot setting. It also outperforms MMNet, the best performing model on COCO-20^*i*^, by 1.2% in the 1-shot setting and RePRI, the best performing model, by 0.6% in the 5-shot setting. The experimental results demonstrate that our model can perform equally well in more difficult scenarios.

**Table 3:** Comparison with state-of-the-arts on COCO-20^*i*^ dataset under **1-shot** and **5-shot** settings.

**Method**	**Backbone**	**1-shot**					**5-shot**				
		**Fold-0**	**Fold-1**	**Fold-2**	**Fold-3**	**Mean**	**Fold-0**	**Fold-1**	**Fold-2**	**Fold-3**	**Mean**
FWB (Nguyen et al., 2019)	VGG16	18.4	16.7	19.6	25.4	20.0	20.9	19.2	21.9	28.4	22.6
PFENet (Tian et al., 2020)	VGG16	33.4	36.0	34.1	32.8	34.1	35.9	40.7	38.1	36.1	37.7
SAGNN (Xie et al., 2021)	VGG16	35.0	40.5	37.6	36.0	37.3	37.2	45.2	40.4	40.0	40.7
RePRI (Boudiaf et al., 2021)	ResNet50	31.2	38.1	33.3	33.0	34.0	38.5	46.2	40.0	43.6	42.1
MMNet (Wu et al., 2021)	ResNet50	34.9	**41.0**	37.2	37.0	37.5	37.0	40.3	39.3	36.0	38.2
SCL (Zhang et al., 2021)	ResNet101	36.4	38.6	37.5	35.4	37.0	38.9	40.5	41.5	38.7	39.9
MANet (Ao et al., 2022)	ResNet50	33.9	40.6	35.7	35.2	36.4	39.1	**48.3**	41.1	40.9	42.3
CRCNet (Liu et al., 2022)	ResNet50	35.1	42.2	41.3	36.4	38.7	**40.5**	45.6	42.4	41.2	42.4
**RPMMs (Baseline) (Yang et al.**, **2020****)**	ResNet50	29.5	36.8	28.9	27.0	30.6	33.8	42.0	33.0	33.3	35.5
**DPMC+DAAConv (Ours)**	VGG16	33.1	38.4	35.8	31.5	34.7	37.5	43.1	38.4	42.2	40.3
**DPMC+DAAConv (Ours)**	ResNet50	**36.9**	40.9	**39.1**	**37.9**	**38.7**	39.5	44.5	**42.6**	**44.2**	**42.7**

mIoU of each fold and averaged mIoU of all folds are reported. The baseline is RPMMs. The bold values represent the best performance.

### 4.4. Ablation study

To evaluate the effectiveness of our constructed DPMC and the usefulness of DAAConv in duplex mode, we conducted a series of ablation experiments, as shown in Table 4.

**Table 4:** Ablation study of our DPMC and DAAConv on PASCAL-5^*i*^.

**PMMs**	**DPMC**	**DAA**	**DAAConv**	**mIoU**
✓				55.3
	✓			57.9
	✓	✓		60.2
	✓		✓	**61.9**

PMMs represent the baseline, DPMC represents the complete duplex network we built, DAA represents a single-layer DAAConv using only SE and self-attention layer, and DAAConv represents our complete attention module. The bold values represent the best performance.

#### 4.4.1. Superiority of DPMC

According to two separate experiments conducted by PMMs and DPMC, our designed DPMC effectively improved PMMs. The segmentation accuracy of DPMC has been improved by 2.3% relative to PMMs, thus providing additional evidence that our DPMC design effectively utilizes information from support and query features to enhance image segmentation.

#### 4.4.2. Effectiveness of DAAConv

We evaluated the segmentation results of two experiments: DPMC running alone and DPMC and DAAConv running together. Our findings indicate that the addition of DAAConv can improve segmentation accuracy by 4% in the duplex mode. This experimental result effectively demonstrates the effectiveness of our constructed hybrid attention mechanism in improving the performance of duplex mode in small sample segmentation tasks.

#### 4.4.3. Necessity of double-layer attention structure

We conducted two experiments using DPMC with DAA (DAAConv without Conv and SAM) and DPMC with DAAConv. Our findings indicate that the SAM and Conv layers in DAAConv play a crucial role in enhancing the model's final segmentation accuracy by 1.7%.

#### 4.4.4. Generalization of DAAConv

DAAConv is effective in several prototype models, including CANet, FWB, and PANet. When inserted after the backbone network of these models, DAAConv has improved their performance to some extent, as shown in Table 5.

**Table 5:** Generalization ability of the proposed DAAConv.

**Methods**	**backbone**	**mIoU**	**Improvement**
CANet (Zhang et al., 2019)	ResNet50	55.40	–
CANet+DAAConv	ResNet50	57.28	+1.88
FWB (Nguyen et al., 2019)	ResNet101	55.71	–
FWB+DAAConv	ResNet101	57.22	+1.51
PANet (Wang et al., 2019)	VGG16	48.10	–
PANet+DAAConv	VGG16	50.29	+2.19

## 5. Conclusion and future work

We propose a DAAconv and a DPMC based on duplex mode to solve challenging few-shot segmentation tasks. DAAConv can effectively obtain contextual information and focus on important regions, and the double-layer structure achieves a balance between long-range and close-range information. DAAConv fits well with the idea of focus and suppression of duplex network, which can effectively improve the performance of duplex mode. Meanwhile, DPMC improves the duplex strategy by fully exploiting the information in support and query and fully realizing the support–query interaction. Moreover, DPMC retains the advantages of duplex mode, which can effectively solve complex segmentation scenarios, such as ambiguous boundaries, when combined with DAAConv. Extensive experiments have shown that the combination of DAAConv and DPMC performs well in few-shot segmentation tasks.

Future work will focus on two parts. First, we will continue to improve our model as we attempt to test it on a larger dataset and continuously test it in complex real-world scenarios. Second, we will combine the algorithm with the robotics algorithm to complete a complete set of work from recognition to operation.

## Data availability statement

Publicly available datasets were analyzed in this study. This data can be found at: http://host.robots.ox.ac.uk/pascal/VOC.

## Author contributions

SZ: software, writing-review and editing, and writing-original draft. JY: software, conceptualization, and methodology. WL: supervision. YR: software. All authors have read and agreed to the published version of the manuscript.
